# Shear-Induced Evolution of Cellular Structure and Thermo-Mechanical Properties in PLA/HNC and In-situ Fibrillated PLA/PTFE Composite Foams

**DOI:** 10.1021/acs.langmuir.6c03508

**Published:** 2026-08-27

**Authors:** Silla George Raju, Ramin Hosseinnezhad, Andrzej Galeski

**Affiliations:** † BioMedChem Doctoral School of the University of Lodz and Lodz Institutes of the Polish Academy of Sciences, 21/23 Matejki Street, 90-237 Lodz, Poland; ‡ Centre of Molecular and Macromolecular Studies, Polish Academy of Sciences, Sienkiewicza 112, 90-363 Lodz, Poland

## Abstract

High-performance cellular biopolymeric foams require precise control of processing and filler-induced microstructure. In this study, poly­(lactic acid) (PLA) composite foams with halloysite nanoclay (HNC) and polytetrafluoroethylene (PTFE) were produced via twin-screw extrusion foaming using azodicarbonamide. The effect of screw speed (20–120 rpm) on morphology, crystallization, and compressive properties was investigated. In PLA/HNC, increasing screw speed improved filler dispersion and heterogeneous nucleation, reducing cell size by ∼50–60% and yielding a maximum void fraction of ∼20.5% at 60 rpm. In contrast, PLA/PTFE exhibited complete in situ fibrillation into a three-dimensional nanofibrillar network (∼100–500 nm), whose density increased continuously with screw speed without saturation. PLA/PTFE showed over a 20-fold reduction in crystallization half-time of PLA at 130 °C (vs ∼4-fold for HNC) due to its high nucleation surface area. It also achieved superior compressive performance (∼68–70 MPa·cm^3^/g strength and ∼11 J/g toughness at 120 rpm), exceeding PLA/HNC by ∼50% and ∼80%, respectively, through fibril-driven reinforcement mechanisms of cell walls.

## Graphical abstract

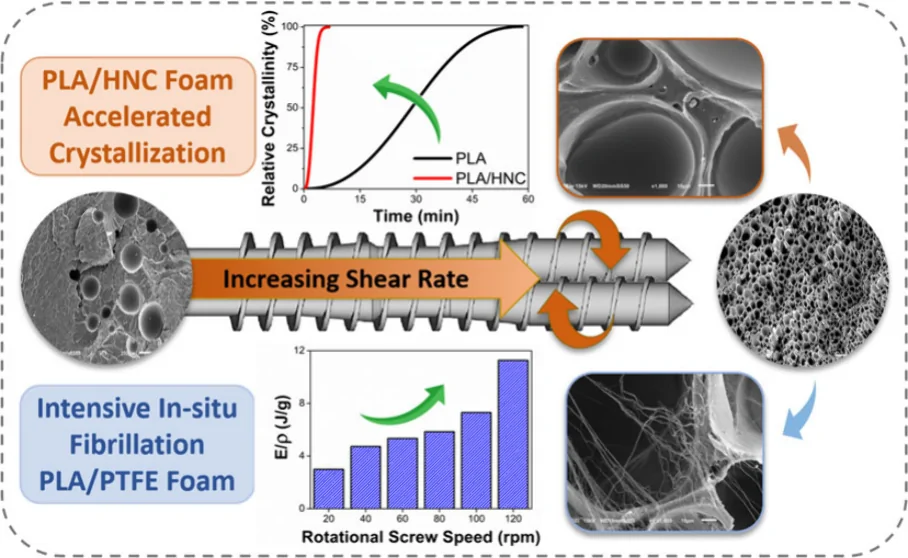

## Introduction

Polymeric foams constitute a structurally and functionally distinct class of materials for a broad range of lightweight applications.^1,2^ The cellular architecture reduces component mass while concurrently introducing attributes including compressive energy absorption, thermal insulation, and acoustic damping.^3–5^ The combination of the cellular architecture with the enhanced matrix properties in so-called polymer nanocomposite foams has attracted growing interest, owing to the synergistic benefits of specific mechanical performance, barrier properties, and functional versatility.^6,7^ Nevertheless, the design of polymer nanocomposite foams is considerably more complex than conventional foams composed of neat polymers. It is widely recognized that nanoparticle-based systems, when incorporated into a medium, can act as antifoaming agents rather than foam promoters.^8^ For instance, montmorillonite platelets function as cell openers, ultimately leading to foam collapse.^9^ However, the underlying causes of these failures have been elucidated. It has been proposed that the successful foaming of polymer nanocomposites is contingent upon the simultaneous satisfaction of three interdependent conditions: fine and uniform dispersion of nanoparticles within the polymer matrix, ensuring smooth bubble wall expansion and formation of mechanically robust struts; adequate adhesion between the nanofiller and the matrix to provide additional heterogeneous nucleation sites without inducing premature cell opening; and the exhibition of strain hardening in the elongational viscosity of the nanocomposite melt, which restricts excessive bubble wall expansion, prevents cell coalescence and collapse, and allows sufficient time for foam stabilization through crystallization during cooling.^10–12^

Biopolymer foams have attracted considerable research interest as environmentally sustainable alternatives to conventional petroleum-based polymer foams. Among the various available biopolymers, PLA has emerged as one of the most compelling candidates for nanocomposite foam development.^13^ Nevertheless, the applications are constrained by well-recognized limitations of PLA: inherently slow crystallization kinetics, which compromise cell wall solidification rates and dimensional stability during processing; low melt strength and extensional viscosity that render the expanding foam susceptible to cell wall thinning, rupture, and coalescence; and the absence of strain hardening in the elongational viscosity response, which precludes the self-limiting bubble growth stabilization mechanism.^14^ There were efforts to successfully modify PLA by chemical modification or low molecular additions. Two main strategies have been identified to overcome these limitations. The first involves the incorporation of carbonaceous materials,^15–17^ organic additives,^18–28^ and inorganic nanoparticles, namely montmorillonite,^29^ nanotalc,^30,31^ and TiO_2_.^32^ Among these fillers, halloysites such as HNC are particularly notable, as they can act as heterogeneous nucleating agents for both bubble initiation during foam expansion and spherulite formation during crystallization of PLA.^33,34^ The geometric discontinuities and high specific surface area of well-dispersed HNC particles reduce the free energy barriers for nucleation in both contexts, increasing cell density, refining cell size distribution, and modestly accelerating PLA crystallization kinetics.^34^ The efficacy of this approach is contingent upon the dispersion state of HNC within the melt and moderate interfacial adhesion between unmodified HNC and the PLA matrix.^33^

The second approach employs the incorporation of a polymeric minor phase as an in situ fibrillating all-polymer nanofiller.^35,36^ For instance, disentangled PTFE and polyethylene can be readily deformed into long nanofibers by the shear fields generated during melt compounding in a twin-screw extruder.^36,37^ The resulting nanofibrillar network simultaneously satisfies all three requirements for successful nanocomposite foaming identified above: PTFE fibrils are finely dispersed within the matrix at nanoscale dimensions; the physical entanglement of fibrils with the surrounding polymer chains provides intimate interfacial contact; and the fibrillar network imparts pronounced strain hardening to the extensional viscosity of the composite melt.^38^ The extension of this concept to in situ generated PLA-based composites involving polyhydroxyalkanoates,^39^ polyamide,^40^ and poly­(butylene succinate)^41^ as minor fibrous components has revealed that the shear-induced crystallization of the minor phase during extrusion is the key factor for the generation of nanofibrillar inclusions that impart significant strain hardening, necessary for foaming.

Despite the recognized merits of HNC and PTFE as good examples of both approaches, a comparative investigation into shear-induced evolution of the cellular morphology and crystallization within a range of extrusion conditions has yet to be reported. Unresolved questions persist regarding the relative nucleation efficiencies of dispersed HNC particles versus continuous PTFE fibrillar networks in the contexts of both foam expansion and polymer crystallization; the critical shear thresholds at which each filler system achieves its functional optimum and beyond which further processing intensity yields diminishing microstructural returns; and whether the progressive, shear-rate-dependent nature of PTFE fibrillation translates into a continuous, saturation-free improvement in compressive mechanical performance. To our knowledge, this is the first study to directly compare HNC and in situ fibrillating PTFE in the same PLA matrix under identical processing conditions. It identifies the shear thresholds for deagglomeration and fibrillation and evaluates whether the resulting improvements in cellular refinement and compressive properties reach a plateau or continue with increasing shear rate. The present study addresses these gaps through the fabrication and characterization of PLA/HNC and PLA/PTFE composite foams across processing shear rates to establish relationships between screw speed, filler dispersion, and fibrillation state and cellular morphology for both composites. This was also to elucidate the nucleation mechanisms operative in HNC- and PTFE-modified foams and their differential consequences for void fraction development and foam density, as well as to characterize the influence of filler type and processing conditions on PLA crystallization kinetics. The results define the processing–structure–property relationships governing compressive strength and energy absorption, with particular emphasis on progressive PTFE fibrillation in driving continuous mechanical improvement.

## Experimental Section

### Materials

Commercial poly­(lactic acid) (PLA, Ingeo 4032D, NatureWorks LLC, USA), with a specific gravity of 1.24 g/cm^3^ and a melt flow index of 6 g/10 min (210 °C, 2.16 kg), was employed as the matrix material. This semicrystalline grade was selected for its established processability in extrusion-based foam applications. Halloysite nanoclay (HNC) nanopowder, with a density of 2.53 g/cm^3^ and a pore volume in the range of 1.26–1.34 mL/g, was sourced from Sigma-Aldrich. Polytetrafluoroethylene (PTFE) powder flakes with a mean particle size of 28 μm and a melting point of 345.7 °C were supplied by DuPont. The chemical blowing agent (CBA) utilized was azodicarbonamide (ADC, Porofor ADC/L-C2, Lanxess, Germany), an exothermic decomposition agent with a decomposition onset temperature of 214 °C and a volumetric gas yield of 228 mL/g at 210 °C. All materials were used in their received state.

### Preparation of Composite Foams

Prior to processing, all constituent materials were dried in a convection oven at 60 °C for 24 h to avoid hydrolytic degradation during melt processing. Composites were prepared at fixed weight ratios of PLA/HNC (99:1 wt %) and PLA/PTFE (95:5 wt %), with ADC loading maintained constant at 3 phr across all formulations.^33^ The compositions used for each filler system (1 wt % HNC and 5 wt % PTFE) correspond to the optimized formulations, in which HNC and PTFE contents were independently varied to establish the best combination of cellular uniformity and mechanical retention. Five wt % of PTFE was found to be essential to exceed the fibril–fibril percolation threshold before a continuous reinforcing network can form.^33^

Prior to extrusion, the constituent powders and pellets were premixed to achieve a homogeneous blend. Foam extrusion was carried out using a corotating twin-screw extruder (2 × 20/40D EHP, Zamak Mercator, Poland) operating under a sequential temperature profile of 180, 185, 190, 195, 200, 200, 210, 210, 210, and 210 °C from the feed zone to the die. The screw rotational speed was varied from 20 to 120 rpm in increments of 20 rpm to investigate shear-induced evolution of the composite foam microstructure and properties, while all other processing parameters were held constant. The calculated apparent shear rates corresponding to the investigated screw speeds are presented in Table S1.

### Characterization

#### Morphological Analysis

The surface morphology of the foamed samples was examined using a scanning electron microscope (SEM, JEOL JSM-5500 LV, Japan). Internal foam cross sections were exposed by cryogenic fracture in liquid nitrogen to preserve the cellular structure without experiencing plastic deformation. The exposed surface of the sample was coated with a gold layer using ion sputtering (JEOL JFC-1200, Japan) prior to analysis. Cell size distributions were quantitatively analyzed from SEM micrographs using ImageJ software.

The densities of unfoamed and foamed samples were ascertained using a density balance (GRAM FD410, Spain) by measuring the weights of a minimum of five samples. The void fraction (*V*_f_) and foam expansion ratio (ϕ) were calculated using the following equations:

1 $$V_{f}=\left(\frac{\rho_{s}-\rho_{f}}{\rho_{s}}\right)\times 100$$

2 $$\phi =\frac{\rho_{s}}{\rho_{f}}$$

where ρ_s_ and ρ_f_ are the densities of unfoamed and foamed samples, respectively.

The cell-population density (*N*_0_) per unit volume of the foamed samples was calculated from SEM micrographs obtained, using the equation:

3 $$N_{0}={\left(\frac{n}{A}\right)}^{3/2}\left(\phi \right)$$

where *n* represents the number of cells and *A* denotes the area of the SEM micrograph, respectively.

For each processing condition, cell size distributions were quantified from five SEM micrographs acquired from at least two independent fracture surfaces per sample, with a minimum of 60 cells analyzed per condition; the mean cell diameter (*D*_av_) is reported in the figures.

#### Rheological Experiments

The melt rheological behavior of neat PLA and the composite was characterized using a strain-controlled rotational rheometer (ARES LS2, TA Instruments, USA) in steady-state shear mode. Measurements were performed at 180 °C using cone-and-plate geometry with a cone angle of 0.1 rad and a gap of 1 mm. Disk-shaped specimens were prepared from 1 mm thick compression-molded films. Steady-state viscosity was measured over a shear rate range of 1 to 1000 s^–1^ to capture the full shear-thinning response relevant to twin-screw extrusion processing conditions.

#### Thermal Analysis

Thermal analysis was performed using a differential scanning calorimeter (DSC Q20, TA Instruments, USA) under a dry nitrogen purge flow rate of 50 mL/min. Specimens of 6–10 mg mass were encapsulated in standard aluminum pans. A three-stage thermal program was employed at 10 °C/min: an initial heating ramp from 40 to 200 °C to characterize the as-processed thermal state; cooling from 200 to 40 °C to assess nonisothermal crystallization behavior; and a second heating ramp to 200 °C to evaluate the crystalline microstructure developed during the controlled cooling cycle. Isothermal crystallization experiments were conducted by cooling specimens from the melt to target crystallization temperatures (*T*_c_) of 110 and 130 °C and maintaining isothermal conditions until crystallization was complete to determine half-crystallization time. The degree of crystallinity (χ_c_) was calculated from the measured enthalpy values according to

4 $$\chi_{c}\left(\%\right)=\left(\frac{\Delta H_{m}-\Delta H_{\mathrm{cc}}}{\Delta H_{m}^{0}\times \left(1-w_{t}\right)}\right)$$

where Δ*H*_m_ and Δ*H*_cc_ are the measured melting enthalpy and cold crystallization enthalpy, respectively, and 93.6 J/g denotes the theoretical melting enthalpy of 100% crystalline PLA.^33^

#### Mechanical Properties

Compression tests of the foam composites were performed using a universal testing machine (Instron 5582, USA). Cylindrical samples measuring 8 × 4 mm (length × diameter) were subjected to axial compression using a 100 kN load cell at a rate of 5% of the initial length per minute. A minimum of five specimens per condition were tested. The specific yield strength (σ_*y*_/ρ) and specific toughness (*E*/ρ) were calculated by integration of the stress–strain curve up to 50% compressive strain, normalized by specimen density, to enable density-independent comparison of mechanical performance.

## Results and Discussion

### Rheological Properties

The melt rheological behavior of neat PLA and its nanocomposites was investigated to elucidate the role of viscoelastic properties in governing foamability. The temperature- and shear-dependent rheological responses are summarized in Figure 1a–d. The evolution of storage modulus (*G*′) and loss modulus (*G*″) with temperature is presented in Figure 1a. For both neat PLA and its composites, both moduli decrease progressively from 180 to 210 °C, reflecting the expected reduction in melt elasticity and viscosity with increasing temperature. At 180 °C, neat PLA exhibits a *G*′ of approximately 5000 Pa, which decreases to ∼1100 Pa at 210 °C (∼78% reduction). The incorporation of HNC markedly lowers the elastic modulus across the entire temperature range, with *G*′ decreasing from ∼3200 Pa at 180 °C to below 400 Pa at 210 °C. In contrast, the PLA/PTFE composite maintains comparatively higher modulus values than PLA/HNC at elevated temperatures, with *G*′ remaining around 1100 Pa at 210 °C. This indicates improved elastic stability of the PTFE-modified system at processing temperatures relevant to foaming.

**Figure 1:**
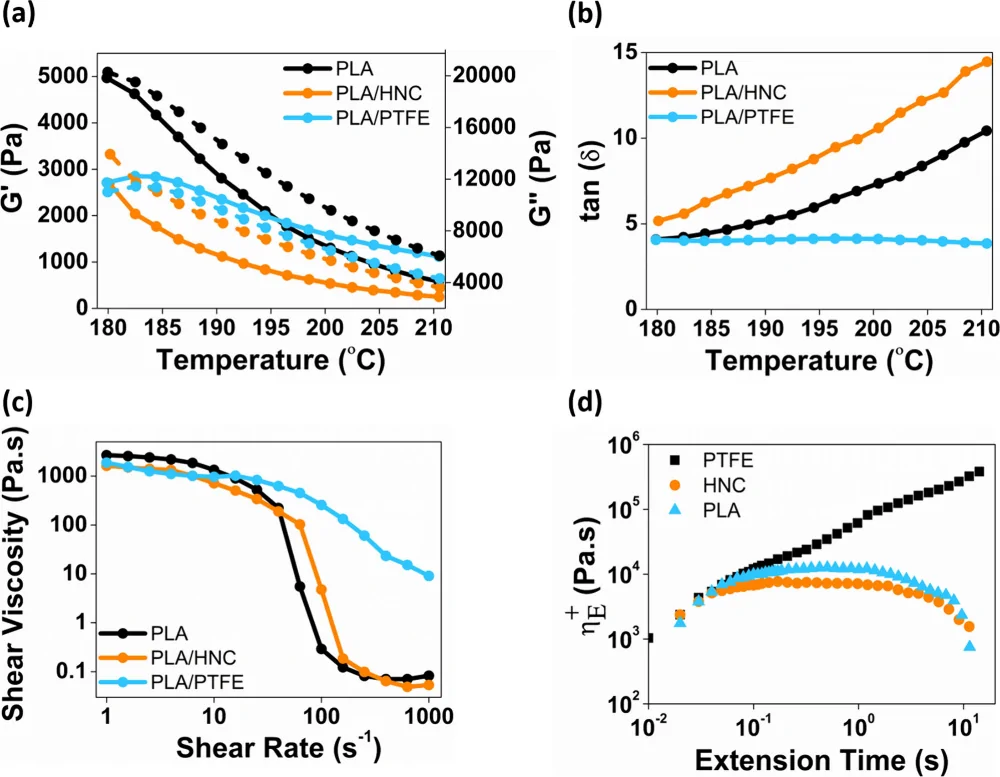
(a) Temperature dependence of storage modulus (*G*′, solid lines, left axis) and loss modulus (*G*″, dashed lines, right axis) for neat PLA, PLA/HNC, and PLA/PTFE, (b) Tan δ as a function of temperature, (c) Shear viscosity as a function of shear rate at 180 °C, (d) Extensional viscosity as a function of extension time at Hencky strain rate of 0.1 s^–1^.

The tan δ (*G*″/*G*′) evolution with temperature (Figure 1b) better clarifies the above discussion. Neat PLA shows a steady increase in tan δ from ∼4 at 180 °C to above 10 at 210 °C, indicating increasing viscous dominance at higher temperatures. The PLA/HNC system exhibits even higher tan δ values, rising from ∼5 to approximately 14.5 over the same temperature interval, suggesting enhanced viscous dissipation and reduced melt elasticity. Conversely, the PLA/PTFE composite maintains an almost constant tan δ (∼4) across the entire temperature range, with a slight downward trend at high temperatures. This behavior indicates a significantly enhanced elastic contribution relative to the viscous component, particularly under thermal softening conditions. The suppression of tan δ growth at elevated temperatures suggests that PTFE restricts molecular mobility and stabilizes the elastic network within the melt.

The temperature dependence of complex viscosity (η*) is shown in Figure S1. Neat PLA exhibits a monotonic decrease from approximately 2100 Pa·s at 180 °C to ∼600 Pa·s at 210 °C. The PLA/HNC composite shows consistently lower viscosity values across the entire temperature range, decreasing from ∼1400 Pa·s to ∼350 Pa·s, indicating a plasticizing effect of HNC on the PLA melt. In contrast, PLA/PTFE maintains intermediate viscosity values between neat PLA and PLA/HNC. The reduced complex viscosity and moduli of the composites relative to neat PLA could be attributed to the lubricating effect of PTFE and HNC, which have been reported to enhance chain mobility and slippage.^42,43^ This finding is consistent with the enhanced elastic character of PLA/PTFE observed in tan δ.

The steady shear viscosity as a function of shear rate at 180 °C is presented in Figure 1c. Neat PLA exhibits pronounced shear-thinning behavior, reflecting disentanglement and chain alignment under flow. The PLA/HNC composite follows a similar shear-thinning trend, with slightly increased viscosity across the middle range of shear rates. The effect of PTFE is markedly different. While the low-shear viscosity (1–10 s^–1^) remains comparable to neat PLA (∼1200–1500 Pa·s), the PLA/PTFE composite maintains significantly higher viscosity at intermediate and high shear rates (100–1000 s^–1^), remaining in the range of ∼10–100 Pa·s where neat PLA and PLA/HNC drop to near 0.1 Pa·s. This corresponds to an increase of approximately two to 3 orders of magnitude in viscosity, indicating the formation of a melt-strengthening microstructure in the presence of PTFE. The fibrillation of PTFE under shear generates physical networks, leading to restricted chain mobility and retarded flow-induced relaxation.^43^ The transient extensional viscosity (η_*E*_^+^) at a Hencky strain rate of 0.1 s^–1^ is shown in Figure 1d. Neat PLA exhibits a modest initial rise in η_*E*_^+^ followed by a decline at longer extension times. PLA/HNC follows a similar response, indicating that discrete HNC particles do not significantly hinder chain disentanglement during uniaxial extension. In contrast, PLA/PTFE displays a continuous, nearly two-orders-of-magnitude increase in η_*E*_^+^ with no maximum, confirming that the in situ PTFE fibrillar network produces pronounced strain hardening to the PLA melt.

### Thermal Properties and Crystallization Kinetics

The thermal behavior of neat PLA and its HNC and PTFE composites is presented in Figure 2. The first heating thermograms (Figure S2) reflect that neither HNC nor PTFE incorporation substantially perturbs the segmental mobility of the PLA amorphous phase or shifts the glass transition (*T*_g_) in the vicinity of 58–60 °C appreciably. Above *T*_g_, all specimens display a cold crystallization exotherm. All three materials display a melting endotherm in the 165–168 °C range (Table 1), with the melting temperature (*T*_m_) of the composites shifted slightly to higher values, consistent with the formation of more thermally stable lamellae.

**Figure 2:**
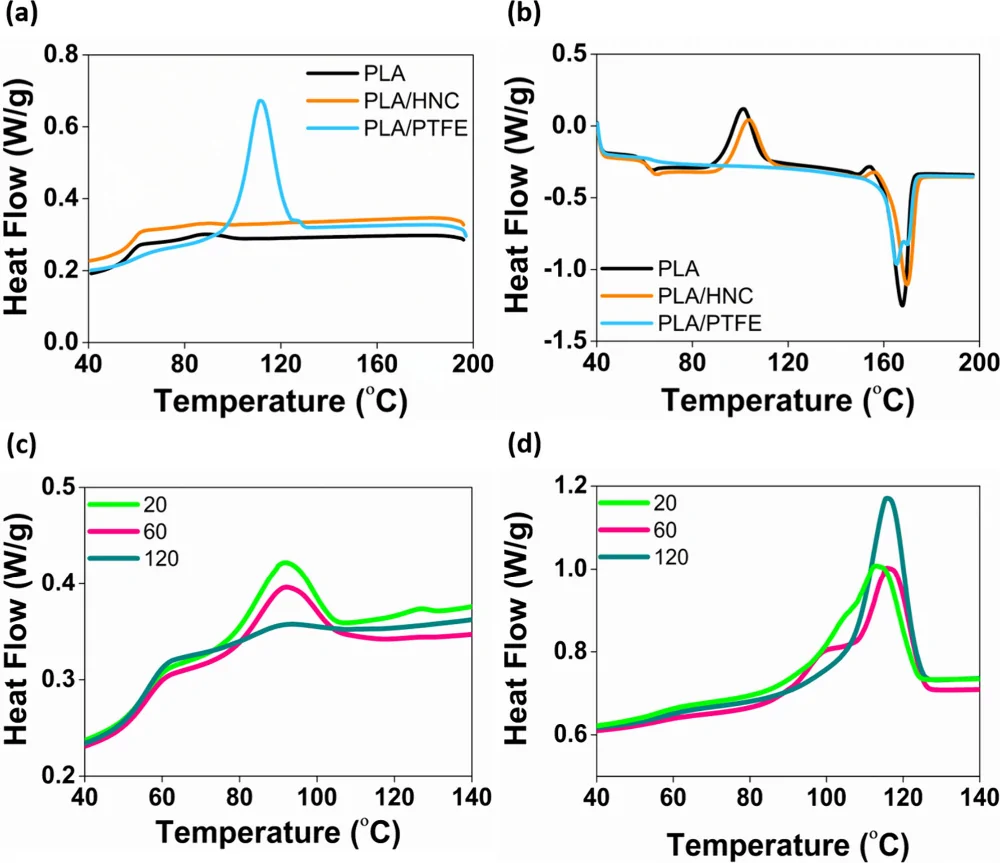
DSC thermograms of neat PLA, PLA/HNC, and PLA/PTFE composites: (a) cooling from the melt; (b) second heating scan. Cooling scans of (c) PLA/HNC and (d) PLA/PTFE foams processed at 20, 60, and 120 rpm.

**Table 1:** DSC Data of Neat PLA and Its Composites upon First and Second Heating Cycles

	first cycle						second cycle				
sample	*T*_c_ (°C)	Δ*H*_c_ (J/g)	*T*_cc_ (°C)	Δ*H*_cc_ (J/g)	*T*_m_ (°C)	Δ*H*_m_ (J/g)	*T*_cc_ (°C)	Δ*H*_cc_ (J/g)	*T*_m_ (°C)	Δ*H*_m_ (J/g)	χ_c_ (%)
PLA	88.3	1.9	100.5	26.9	169.8	39.7	101.2	27.4	167.8	42.1	15.7
PLA/HNC	89.8	0.9	103.9	23.6	171	30.2	104.4	24.8	169.7	33.4	9.5
PLA/PTFE	111.2	30.8	98.9	23.4	170.9	37.2			165.4	31.9	35.9

The cooling thermograms (Figure 2a) provide the most discriminating evidence of filler-induced modification of PLA crystallization behavior. Neat PLA and the PLA/HNC composite both exhibit broad, low-intensity exothermic signals during cooling from the melt, indicative of sluggish and incomplete crystallization. In contrast, the PLA/PTFE composite displays a sharp, prominent crystallization exotherm with a peak temperature of approximately 108–110 °C and a maximum heat flow approaching ∼ 0.65–0.70 W/g. The intensity is approximately 10-fold greater than that of the HNC composite and neat PLA under equivalent cooling conditions. This proves that the in situ generated PTFE fibrillar network acts as a highly effective nucleating template for PLA crystallization from the melt. The cold crystallization exotherm observed in the second heating scan of neat PLA and HNC composite (Figure 2b) remains prominent, consistent with the limited crystallization observed in Figure 2a. The PLA/PTFE composite, in contrast, shows a markedly suppressed cold crystallization due to the substantially higher degree of crystallinity achieved during the cooling cycle as a consequence of the accelerated crystallization kinetics observed in Figure 2a. The melting endotherms of PLA/PTFE show the characteristic double-peak melting morphology associated with the simultaneous presence of less-perfect crystallites and more stable lamellae. The double-peak melting endotherm observed for PLA/PTFE during the second heating scan is attributed to melt recrystallization. Rapid crystallization induced by the PTFE fibrillar network during cooling produces numerous thin, less-perfect crystallites. Upon reheating, these lamellae partially melt and recrystallize into thicker, more stable crystals before final melting, giving rise to the low- and high-temperature melting peaks, respectively. Cooling scans were also recorded for PLA/HNC and PLA/PTFE foams processed at 20, 60, and 120 rpm. For PLA/HNC (Figure 2c), the crystallization exotherm broadens and its peak intensity decreases above 60 rpm, reflecting HNC deagglomeration. At low screw speeds, crystallization occurs around a few large agglomerates, producing sharp exotherms. Above ∼60 rpm, these agglomerates break into numerous finely dispersed nucleating particles, initiating crystallization at lower supercooling but over a broader temperature range. In contrast, PLA/PTFE (Figure 2d) exhibits exotherms at much higher temperatures (∼110–115 °C vs ∼90–100 °C for PLA/HNC), confirming the superior nucleating efficiency of the fibrillar PTFE network. The exotherm becomes progressively sharper and more intense from 20 to 120 rpm, indicating increasing PTFE fibrillation.

The isothermal crystallization of the samples evaluated at 130 and 110 °C is presented in Figure 3a,b as relative crystallinity versus time curves, with the corresponding half-crystallization times (*t*_1/2_) summarized in Figure 3c. At 130 °C (Figure 3a), PLA exhibits retarded crystallization over a time scale of approximately 130–140 min before reaching completion and a *t*_1/2_ of 68.1 min. The incorporation of HNC reduces *t*_1/2_ dramatically to 12.8 min, representing a 5-fold acceleration relative to neat PLA, and the crystallinity curve reaches saturation within approximately 15–20 min. The PTFE composite demonstrates an even more pronounced kinetic enhancement, with *t*_1/2_ reduced to 2.9 min. This result is over 20-fold acceleration over neat PLA and a further 4-fold improvement relative to the HNC composite.

**Figure 3:**
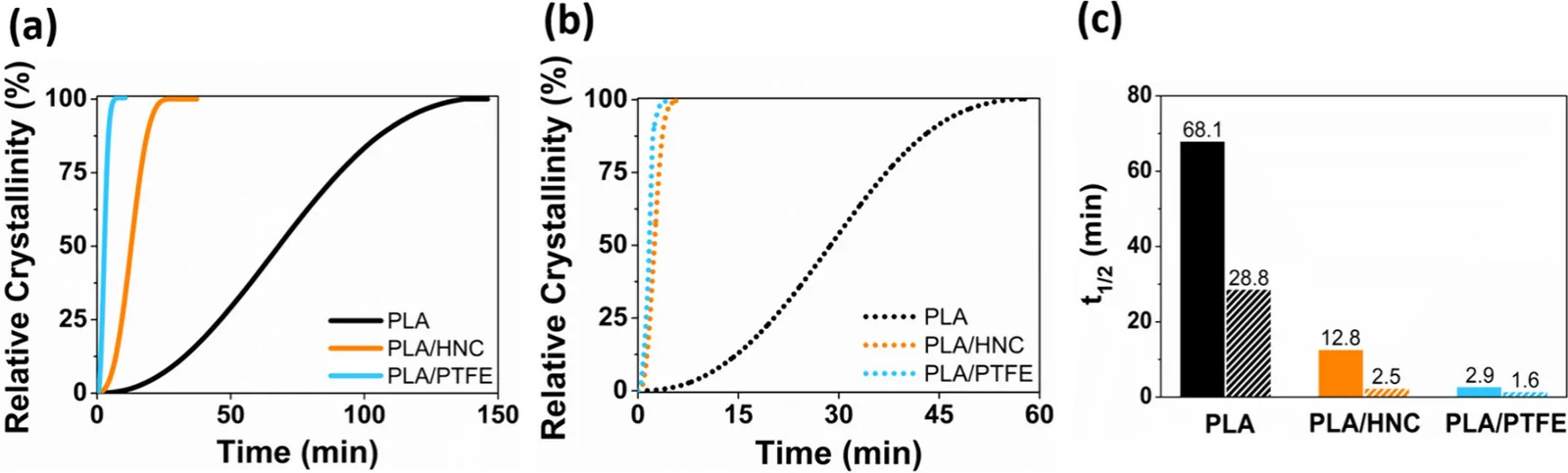
Isothermal crystallization kinetics of neat PLA, PLA/HNC, and PLA/PTFE composites: relative crystallinity as a function of time at (a) 130 °C and (b) 110 °C; (c) half-crystallization time (*t*_1/2_) values extracted from isothermal crystallization curves at 130 °C (solid column) and 110 °C (patterned column).

The crystallization kinetics are substantially accelerated at the lower crystallization temperature of 110 °C (Figure 3b). Under these conditions, the kinetic differentiation between the composites and PLA is even more pronounced. The neat PLA curve at 110 °C, while progressing more rapidly than at 130 °C, remains substantially slower than either composite. PLA/HNC composite achieves a *t*_1/2_ of 2.5 min, more than an order of magnitude reduced relative to PLA, and reaches full crystallinity within approximately 5 min. PLA/PTFE composite attains *t*_1/2_ = 1.6 min at 110 °C, completing the fastest crystallizing within approximately 3 min. The improved crystallization of PLA/PTFE composite is consistent with the morphological evidence. The in situ fibrillated PTFE network, characterized by fibers of ∼600 nm diameter and high aspect ratio intimately entangled with the PLA matrix, provides a spatially continuous and geometrically extensive heterogeneous nucleation substrate. The high specific interfacial area and physical continuity of this fibrillar network enable simultaneous nucleation at a vastly greater number of sites per unit volume compared to the discrete HNC particle dispersion.

### Cellular Morphology of PLA/HNC and PLA/PTFE Foams

The evolution of cellular morphology of PLA/HNC composite foamed with 3 phr azodicarbonamide as a function of screw rotational speed is presented in Figure 4a–f, together with the corresponding cell size distributions. At 20 rpm (Figure 4a), the foam exhibits a coarse, heterogeneous cellular structure with large spherical cells (∼180–200 μm) and smaller cells ranging from 50 to 120 μm. The limited cell population indicates low cell density under a low shear rate. Increasing the speed to 40 rpm (Figure 4b), noticeably refines the cellular structure, shifting it toward smaller diameters (30–100 μm) with a peak around 60–80 μm. Although occasional large cells (>150 μm) remain, their frequency is significantly reduced. At 60 rpm (Figure 4c), the structure becomes finer and more uniform, with cells ranging from 30 to 90 μm. Large cells above 150 μm become marginal, while the increased cell count indicates a substantial rise in cell density. The pronounced evolution is observed at 80 rpm (Figure 4d), where a homogeneous and densely packed cellular architecture with a narrowed cell-size distribution of 20–70 μm is obtained. The cell count is significantly higher, and cells larger than 100 μm become less frequent. At higher speeds of 100 and 120 rpm (Figure 4e,f), the foam exhibits a highly refined structure with a dominant cell size below 60 μm, with a strong concentration of cells between 20 and 50 μm, with very few exceeding 100 μm. The markedly higher cell count suggests a substantial increase in nucleation density. Compared to 20 rpm, the average cell size decreases by ∼50–60%, while the cell density increases several-fold. Applying high shear rate, the number of nucleation sites increases due to the finer dispersion of azodicarbonamide agglomerates. The resulting high nucleation density restricts individual cell growth through spatial confinement and limited gas availability per nucleus, leading to a finer cellular structure. A more uniform distribution of nucleating sites also lowers the critical free energy barrier for bubble nucleation, further increasing nucleation density and reducing average cell size. The reduction in average cell size by more than half relative to 20 rpm confirms the dominance of nucleation over growth.

**Figure 4:**
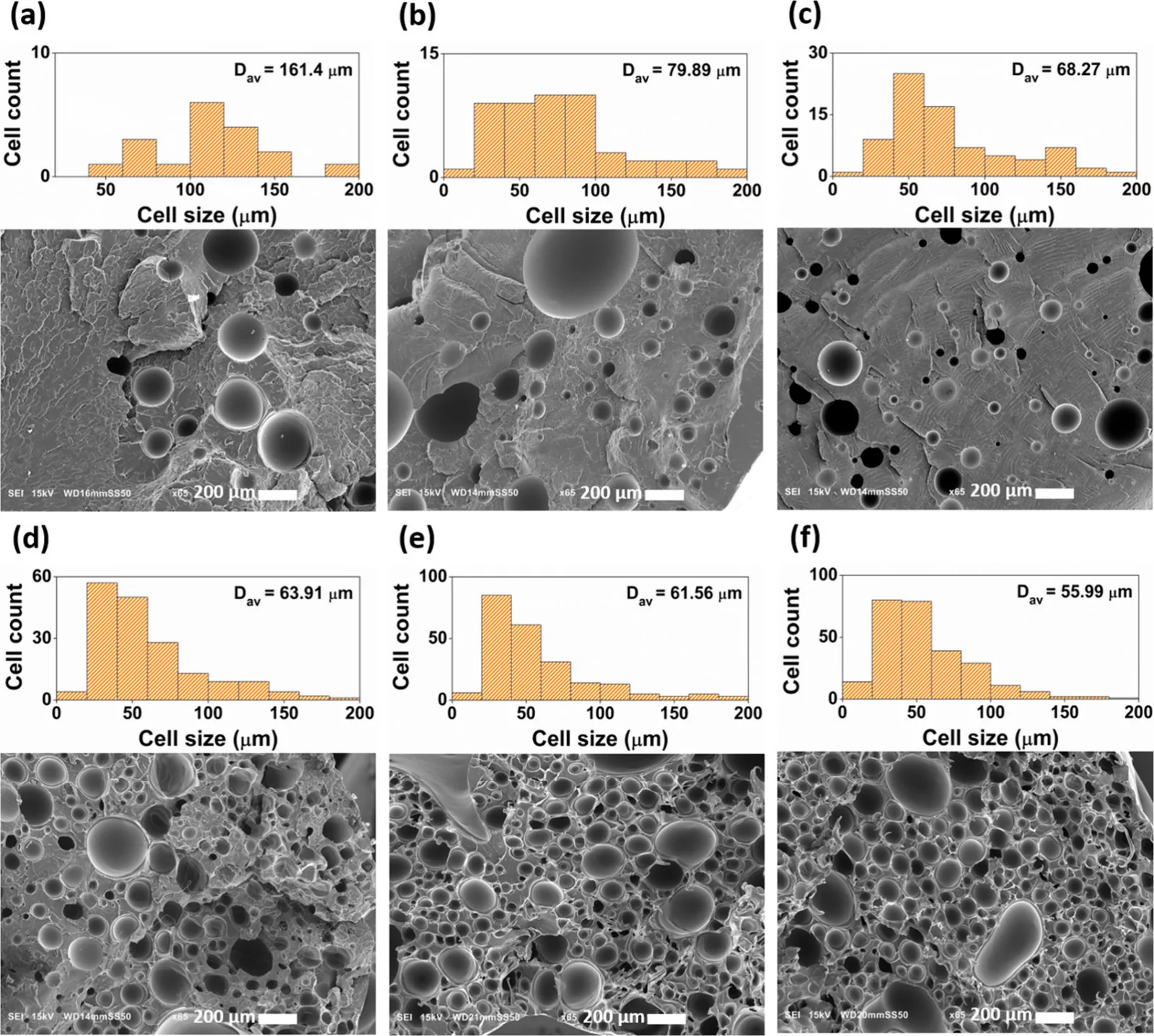
SEM micrographs and corresponding cell size distributions of PLA/HNC composites foamed at (a) 20 rpm, (b) 40 rpm, (c) 60 rpm, (d) 80 rpm, (e) 100 rpm, and (f) 120 rpm. Scale bar: 200 μm.

In the case of PLA/PTFE processed at 20 rpm (Figure 5a), the foam exhibits a sparse and irregular cellular network with a limited number of large, well-separated cells with diameters predominantly in the 100–175 μm range, interspersed with occasional smaller cells below 50 μm. The corresponding histogram confirms a modal bin centered near 75–100 μm and a maximum cell count per bin of only ∼ 10–12. The low cell population and wide size dispersion are consistent with insufficient gas dissolution. At 40 rpm (Figure 5b), a discernible refinement in cellular structure is observed. The distribution shifts toward smaller diameters, with the dominant fraction concentrated between 25 and 75 μm and the peak bin count rising to approximately 15–18. The higher gas yield begins to populate additional nucleation sites, thereby distributing available gas among a greater number of growing cells and restricting individual bubble expansion. At the intermediate condition (Figure 5c), the histogram peak migrates to the 25–50 μm bin, with a maximum cell count approaching ∼28–30, and the upper tail of the distribution contracts noticeably.

**Figure 5:**
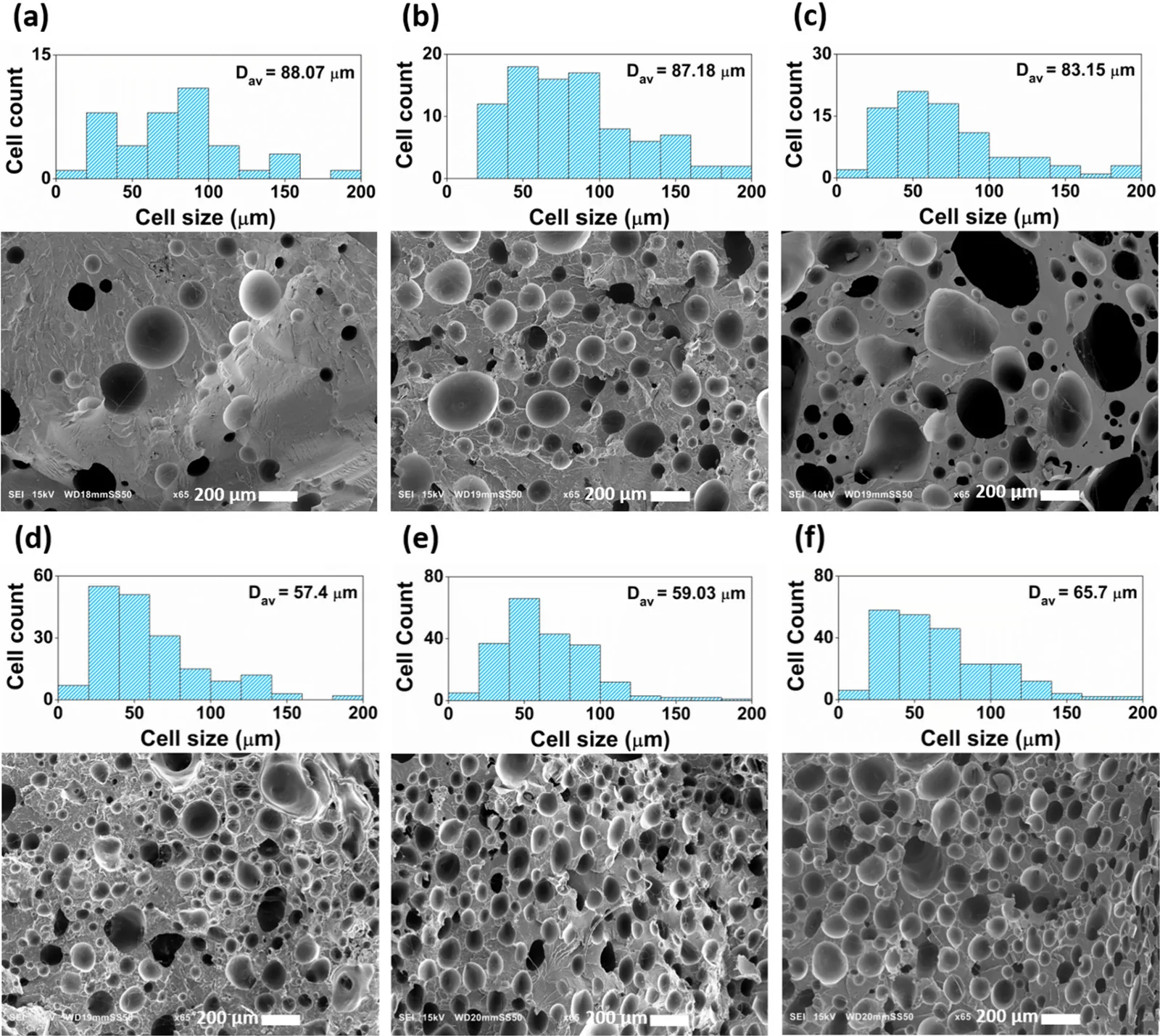
SEM micrographs and corresponding cell size distributions of PLA/PTFE composites foamed at (a) 20 rpm, (b) 40 rpm, (c) 60 rpm, (d) 80 rpm, (e) 100 rpm, and (f) 120 rpm. Scale bar: 200 μm.

The most pronounced microstructural refinement is observed at 80 rpm (Figure 5d). The SEM image reveals a densely packed, highly uniform cellular architecture in which large cells are effectively absent and the cell wall network appears continuous and well-defined. The cell size distribution becomes strongly left-skewed, with the majority of cells concentrated in the 5–50 μm range and the modal bin count rising sharply to ∼55–60. Further increasing the speed (Figure 5e,f) sustains this refined morphology, with histogram peak counts reaching approximately 70–75 and the distribution remaining tightly confined below 75 μm. The corresponding micrograph confirms a high-density foam with predominantly sub-50 μm cells and a notably homogeneous spatial arrangement, suggesting that gas supersaturation is sufficiently high to drive simultaneous, widespread nucleation throughout the melt volume.

### Shear-Induced Morphological Evolutions

The two fillers exhibit contrasting morphologies (Figure 6), relevant to their dispersion behavior and functional roles within the PLA composite matrix. The HNC powder (Figure 6a) displays highly irregular agglomerates with coarse surface topography densely populated with crystalline subparticles of nanometric dimensions. The main agglomerate body, with a lateral dimension on the order of several micrometers, is surrounded by fragmented particles and dispersed fine debris, indicating a loosely consolidated aggregate structure with limited interparticle cohesive strength. This implies that effective dispersion of HNC, providing heterogeneous nucleation for foaming, is critically shear-dependent. Such a shear-induced deagglomeration during extrusion will yield a range of active nucleant sizes, with finer dispersed units offering proportionally greater nucleation efficacy. In contrast, the PTFE particles (∼20–30 μm) exhibit a strikingly regular, smooth-surfaced ovoid morphology (Figure 6b). The absence of surface roughness reflects the nonpolar, low-surface-energy character of PTFE. This morphological characteristic supports the propensity of PTFE to fibrillate during high-shear melt processing, transitioning from discrete ovoid particles into an interconnected fibrillar network.^38^

**Figure 6:**
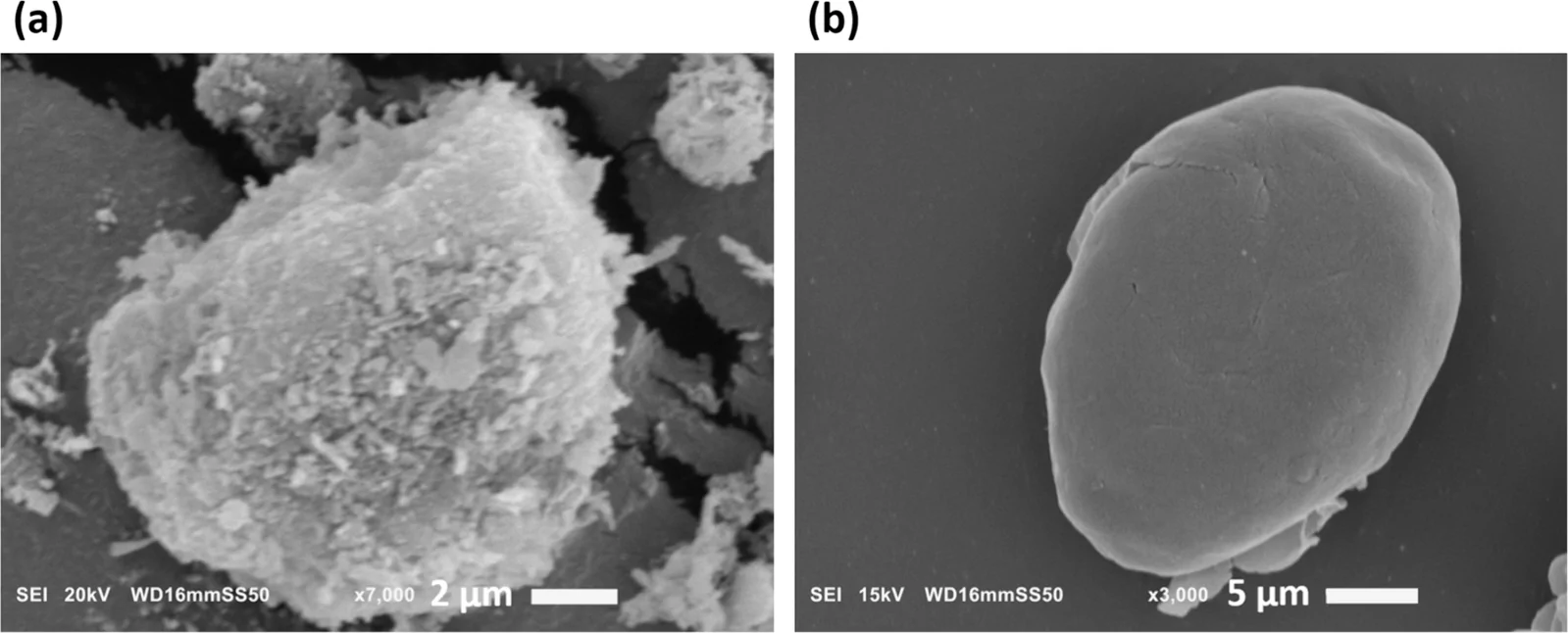
SEM micrographs of (a) HNC particles with rough, crystallite-encrusted surfaces and (b) virgin PTFE particles exhibiting a smooth ovoid morphology.

The internal cellular architecture and filler dispersion state of PLA/HNC and PLA/PTFE composite foams were further examined (Figure 7). The cross-sectional micrograph of the PLA/HNC foam (Figure 7a, left) reveals a well-developed closed-cell structure comprising relatively spherical cells with smooth inner surfaces and intact cell walls. The higher-magnification (Figure 7a, right) resolves HNC particles (∼2 to 5 μm) embedded within the cell wall. While physically incorporated within the PLA cell walls, a number of particles exhibit peripheral interfacial voids. The presence of embedded HNC particles within the cell wall struts implies that they contribute meaningfully to heterogeneous nucleation during foam expansion. However, the observed interfacial debonding suggests that load transfer efficiency between the HNC reinforcement and the PLA matrix under mechanical loading may be constrained. The morphological evolution of PTFE within the PLA matrix is different. Large, elongated cells appeared, within which thin fibrillar structures are already discernible, traversing the cell interior and spanning across adjacent cell walls (Figure 7b). The high magnification confirms the complete in situ fibrillation of the initial PTFE particles, as in Figure 7b, into a well-developed network of nano- and microfibers with individual diameters on the order of ∼ 600 nm. The fibrils are highly elongated, exhibiting significant aspect ratios, and form a physically entangled network that interpenetrates the PLA matrix intimately. Critically, no interfacial voids or debonding features are detectable at the fiber-matrix interface, in contrast to the HNC system. This phenomenon could be attributable to the mechanical interlocking facilitated by effective stress transfer.

**Figure 7:**
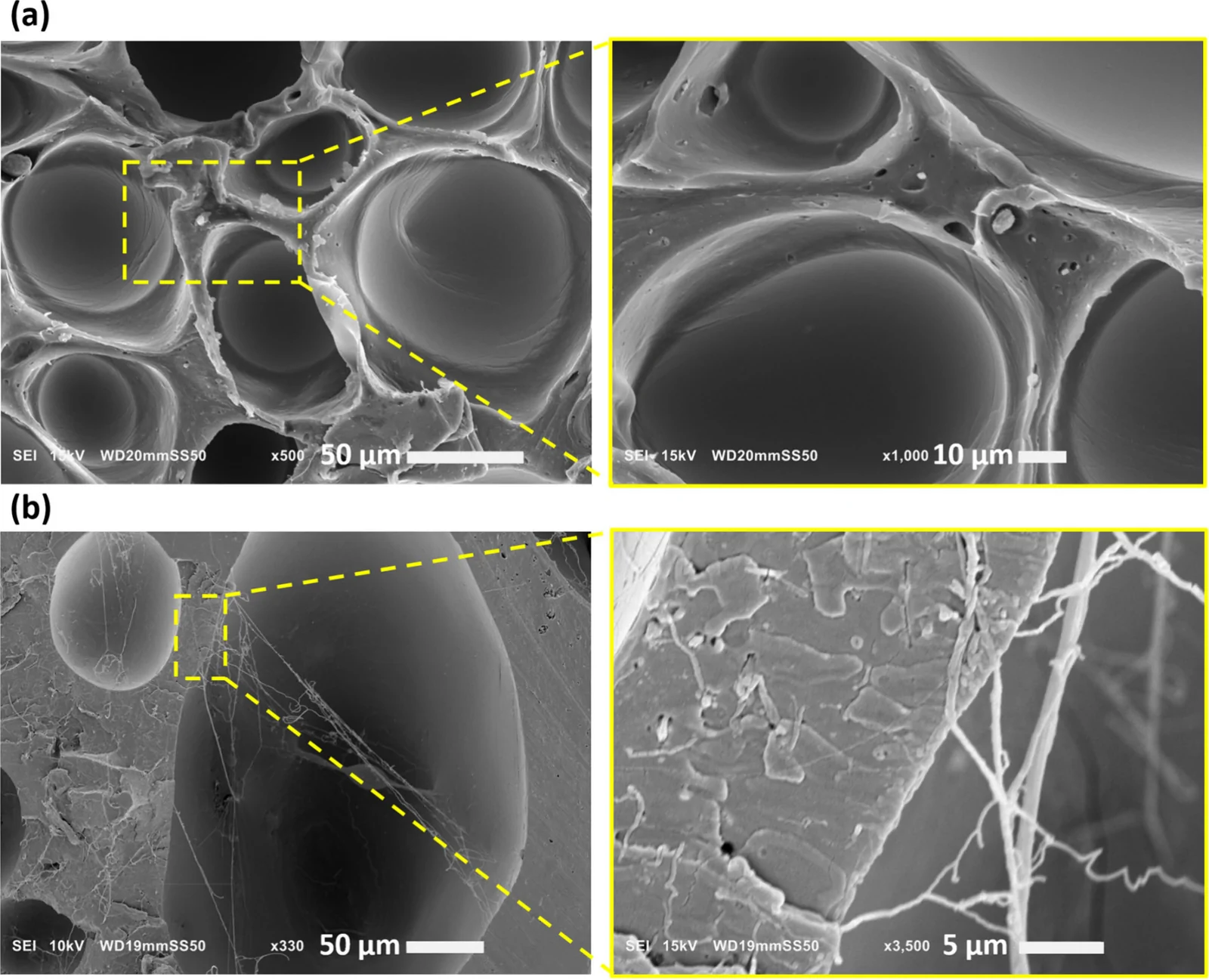
SEM micrographs illustrating filler dispersion and reinforcement morphology in (a) PLA/HNC foam at ×500 (left) and ×1000 (right); (b) PLA/PTFE foam at ×330 (left) and ×3500 (right).

The evolution of void fraction and bulk foam density with increasing rotational speed is presented in Figure 8. As shown in Figure 8a, at the lowest speed of 20 rpm, both composite systems exhibit comparably low void fractions of approximately 9.5% and 8.5% for PLA/PTFE and PLA/HNC, respectively. This reflects the limited shear-induced dispersion of nucleating agents and the insufficient melt homogenization. Under these conditions, poor deagglomeration of HNC particles and incomplete fibrillation of PTFE yield a restricted number of active nucleation sites, confining foam expansion and producing near-solid bulk densities of ∼1.145 g/cm^3^ and ∼1.075 g/cm^3^ for the HNC (Figure 8b) and PTFE (Figure 8c) systems, respectively. With increasing screw speed, the two systems diverge markedly in their foaming response. For the PLA/PTFE system, void fraction rises sharply from ∼9.5% at 20 rpm to ∼16.5% at 40 rpm, an increase of approximately 74%, before reaching a stable plateau. Correspondingly, foam density undergoes a pronounced single-step reduction from ∼1.075 g/cm^3^ at 20 rpm to approximately 0.99 g/cm^3^ at 40 rpm, beyond which it remains invariant (Figure 8c). This abrupt transition suggests that a critical shear threshold is reached at 40 rpm, above which PTFE fibrillation is sufficiently advanced to provide a stable melt-strengthening fibrillar network.

**Figure 8:**
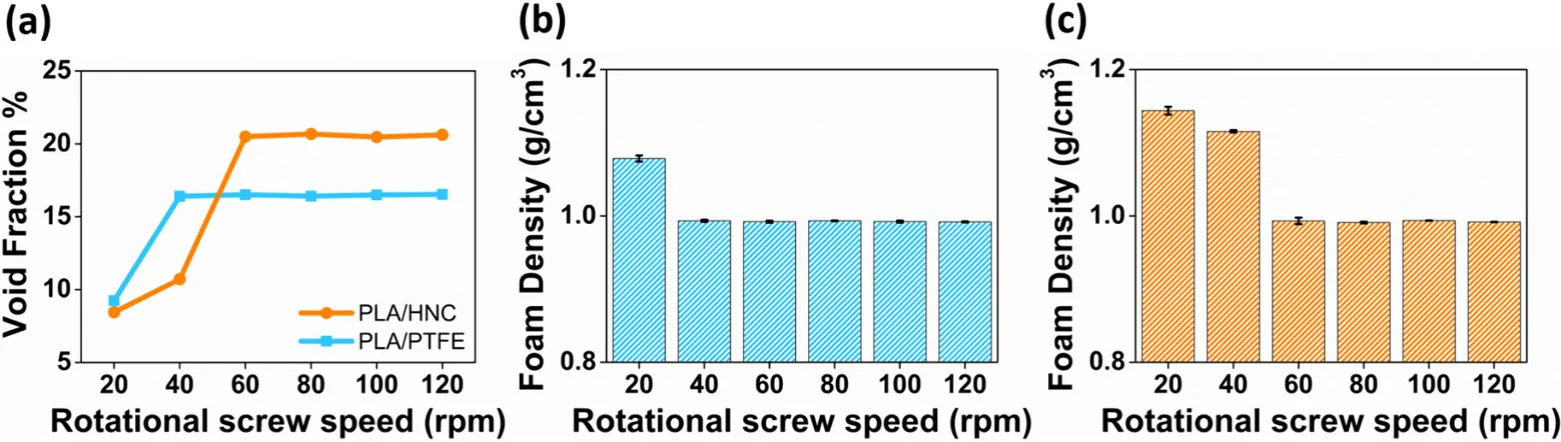
(a) Void fraction as a function of screw rotational speed; foam density as a function of screw rotational speed for (b) PLA/HNC and (c) PLA/PTFE composite foams.

The PLA/HNC system exhibits a distinctly different response. Void fraction increases moderately from ∼8.5% at 20 rpm to ∼11% at 40 rpm, followed by a substantial rise to ∼21% at 60 rpm, at which point it too reaches a plateau sustained through 120 rpm (Figure 8a). The corresponding foam density decreases from ∼1.145 g/cm^3^ at 20 rpm to ∼1.105 g/cm^3^ at 40 rpm, with a more pronounced reduction to ∼0.99 g/cm^3^ at 60 rpm (Figure 8b). The delayed onset of significant void fraction development in the HNC system, requiring 60 rpm to achieve full activation compared to 40 rpm for PTFE, reflects the greater shear energy demand associated with the deagglomeration of HNC particle clusters. Thereby shifting the critical processing threshold to a higher rotational speed.

Interestingly, once respective plateau regimes are attained, the PLA/HNC system achieves a higher ultimate void fraction (∼20.5–21%) compared to the PLA/PTFE system (∼16.5%), despite both systems converging to near-identical foam densities of approximately 0.99 g/cm^3^. This apparent discrepancy is reconcilable by considering the distinct roles of each filler in the foaming process. HNC particles, once adequately dispersed, function primarily as heterogeneous nucleation agents, generating a high density of nucleation sites that promote extensive gas phase formation and cell multiplication.^44^ PTFE fibrils, in contrast, operate principally as a melt rheology modifier, enhancing extensional viscosity and cell wall stability, rather than as a nucleation promoter.^45^ Figure 9 summarizes the shear-induced structural evolution governing the foaming behavior of both systems. In PLA/HNC, shear progressively deagglomerates HNC into a finely dispersed population that promotes heterogeneous nucleation. In PLA/PTFE, the same shear induces PTFE fibrillation, forming an interconnected nano- and microfibrillar network that increases melt viscosity and stabilizes cell walls against thinning and coalescence, consistent with the extensional viscosity (Figure 1d) and compressive results.

**Figure 9:**
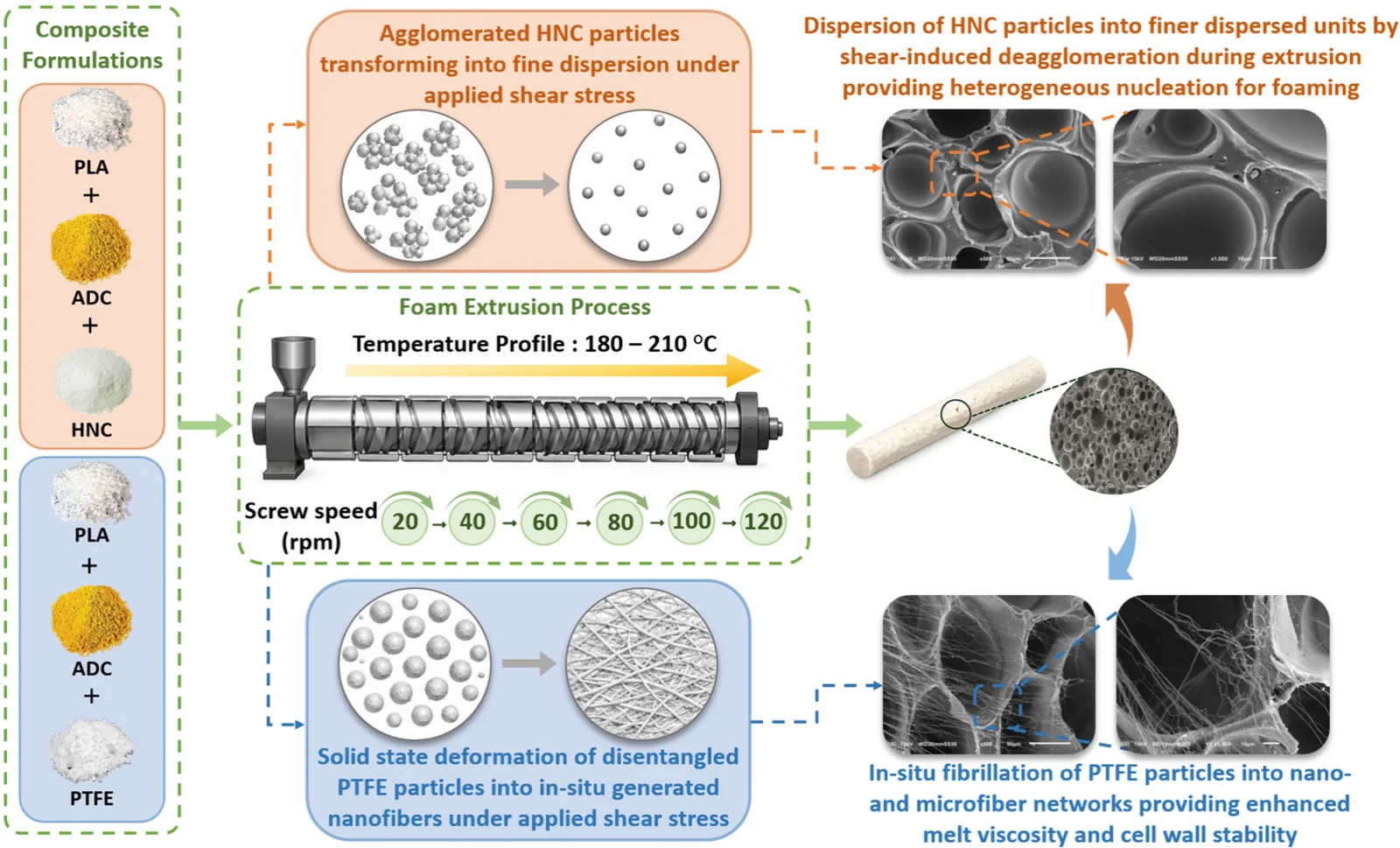
Schematic illustration of the foaming mechanism of PLA composites involving the reinforcement mechanisms of HNC and in situ-generated PTFE nanofibers.

### Compressive Mechanical Properties

The compressive mechanical response of the composite foams processed across rotational speeds is presented in Figure 10. The compressive stress–strain curves of both foams exhibit the characteristic three-stage response of cellular polymer structures: a linear elastic regime, a yield point followed by a stress plateau associated with progressive cell wall buckling and collapse, and an incipient densification at higher strains. The yield stress for PLA/HNC (Figure 10a) at 20 rpm is comparatively low, ∼27 MPa. With increasing screw speed, the yield stress rises progressively, reaching ∼44 MPa at 120 rpm. The specific yield strength (σ_*y*_/ρ) increases ∼75% from 25 MPa·cm^3^/g at 20 rpm to ∼43–45 MPa·cm^3^/g at 120 rpm, and the specific toughness (*E*/ρ) follows a similar trend, rising from ∼3 J/g at 20 rpm to ∼6 J/g at 120 rpm. This confirms that the mechanical improvements are attributable to increased cell density, reduced average cell size, and more homogeneous cellular architecture achieved at higher screw speeds. The behavior of PLA/PTFE foams (Figure 10b) is distinct with a yield stress of ∼35–40 MPa at 20 rpm. With increasing screw speed, the PLA/PTFE foams exhibit pronounced strain-hardening at higher screw speeds. At 120 rpm, the PLA/PTFE foam sustains compressive stresses approaching 135–140 MPa at 50% strain, which is 2.3-fold greater than the corresponding PLA/HNC foam under equivalent deformation and processing conditions. The specific yield strength of the PLA/PTFE increases from ∼25 MPa·cm^3^/g at 20 rpm to ∼68–70 MPa·cm^3^/g at 120 rpm, which is 55% higher than the maximum achieved by the HNC system. The specific toughness similarly demonstrates a continuous increase with screw speed, rising from ∼3 J/g at 20 rpm to approximately 11 J/g at 120 rpm, a near-4-fold enhancement.

**Figure 10:**
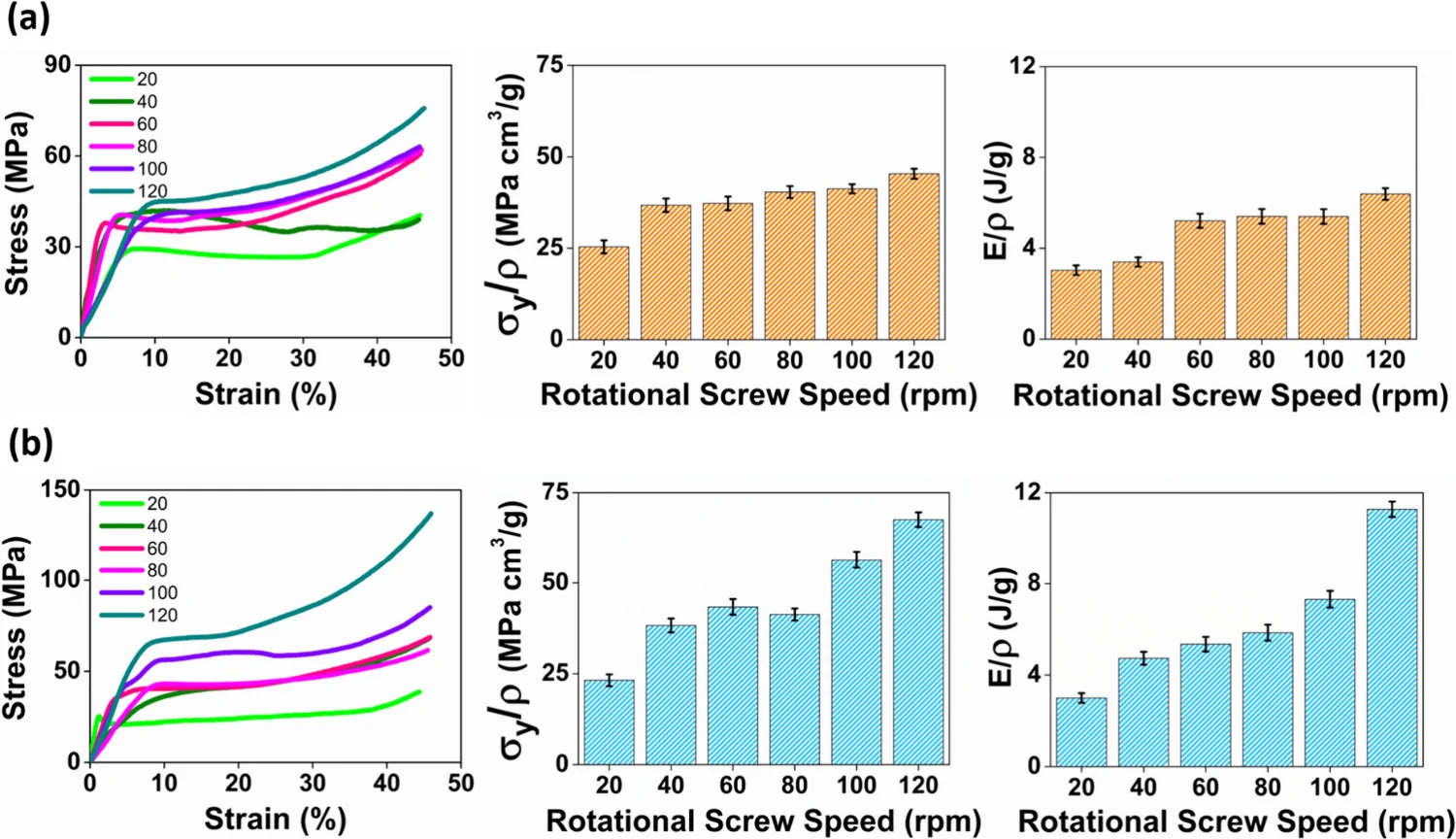
Compressive mechanical properties of PLA composite foams as a function of screw rotational speed: stress–strain curves (left), specific yield strength σ_*y*_/ρ (middle), and specific toughness *E*/ρ (right) for (a) PLA/HNC and (b) PLA/PTFE composite foams processed at 20–120 rpm.

Critically, unlike the HNC system, which exhibits a performance plateau above 80 rpm, PLA/PTFE shows no evidence of saturation, with the largest absolute increments in σ_*y*_/ρ and *E*/ρ observed between 80 and 120 rpm. This is consistent with the progressive advancement of PTFE fibrillation at higher shear rates, generating increasingly fine, extensively entangled fibrillar networks that provide more effective reinforcement of the cell wall architecture. The shear-rate-dependent evolution of PTFE fibrillar morphology within the PLA foam cellular matrix is shown in Figure 11 by SEM micrographs of fracture surfaces from foams processed at 60 and 100 rpm. At 60 rpm (Figure 11a), the foam cell walls and strut surfaces bear a sparse, relatively open fibrillar network. Individual elongated PTFE fibrils are clearly with estimated diameters in the range of approximately 200–500 nm. The fibrils are distributed at relatively low areal density. The substantial interfibril spacing leaves appreciable areas of the cell wall matrix surface unoccupied by fibrillar reinforcement, and the network connectivity remains limited. This morphological state is consistent with a partially fibrillated condition in which the applied shear rate at 60 rpm is sufficient to initiate the transition of PTFE particles from their initial ovoid morphology into elongated fibrillar forms, but insufficient to drive extensive fibril refinement.

**Figure 11:**
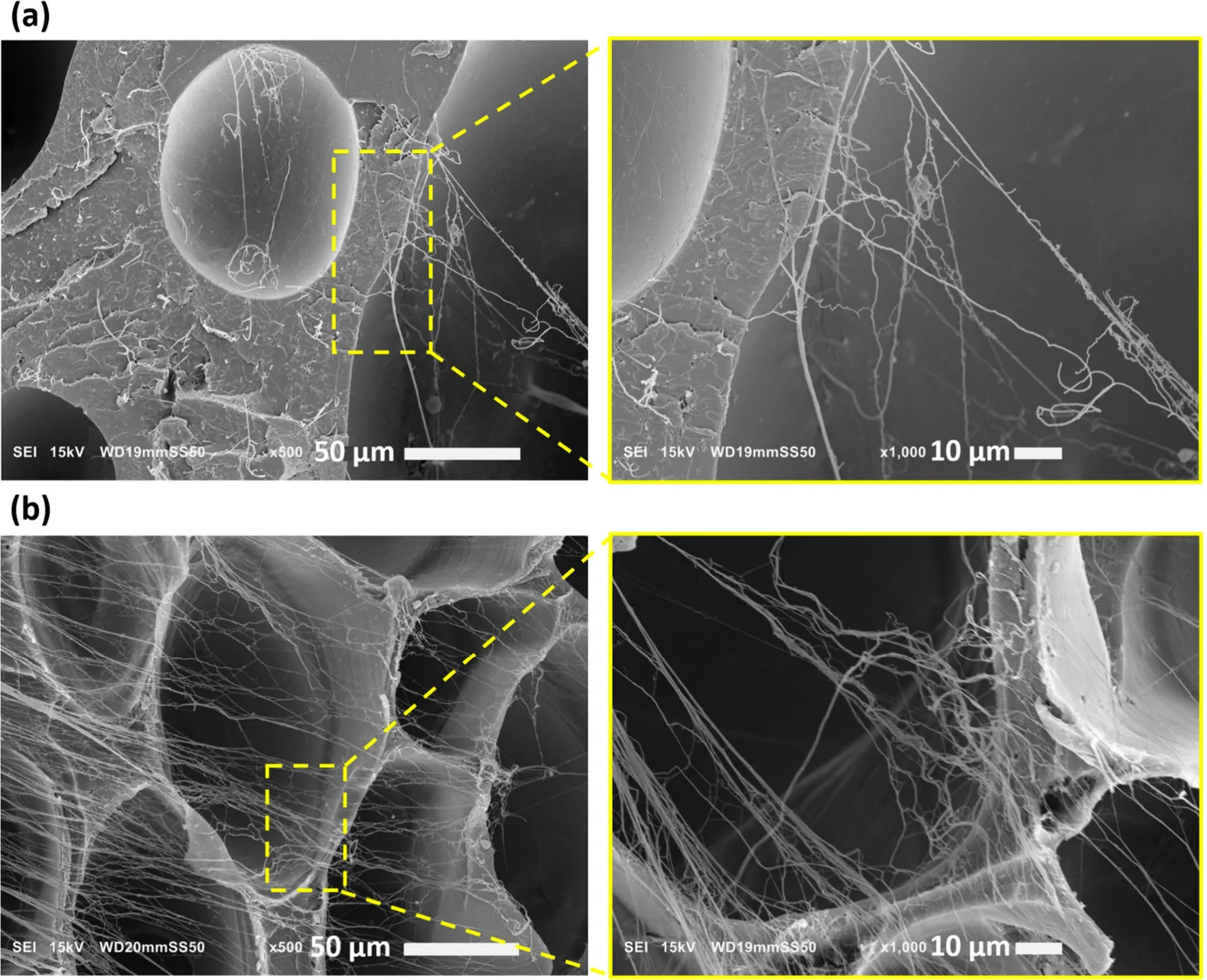
SEM micrographs of fractured PLA/PTFE composite foam cell walls illustrating the shear-rate-dependent evolution of PTFE fibrillar morphology: (a) 60 rpm, limited PTFE fibrils (∼200–500 nm diameter); (b) 100 rpm, entangled PTFE nanofibrillar mesh (∼100–300 nm). The aspect ratio of PTFE fibers is calculated as 160 and 680 for the 60 and 100 rpm, respectively.

In contrast, the PLA/PTFE foam processed at 100 rpm (Figure 11b) exhibits an entangled network comprising fibrils of refined diameter 100–300 nm, interconnected by abundant branching nodes and multifibril junctions. This transition to a dense, extensively entangled three-dimensional network provides direct microstructural evidence reflected in the compressive mechanical behavior (Figure 10b). The fibrillar morphology present at 60 rpm provides a limited reinforcement contribution to the cell walls, σ_*y*_/ρ and *E*/ρ values. As screw speed increases toward 100–120 rpm, the densification and topological connectivity of the PTFE fibrillar network reach a level at which the fibrils form a coherent reinforcing network within and across the cell walls. Under compressive deformation, this scaffold resists cell wall buckling through fibril tensile loading, with continuing increments in σ_*y*_/ρ and *E*/ρ observed between 80 and 120 rpm in Figure 10b. The earlier onset of saturation in the PLA/HNC system reflects the finite nature of agglomerate deagglomeration. Once the HNC agglomerates are broken down to their limiting particle size, no further nucleation surface area can be generated by additional shear. PTFE fibrillation, in contrast, is a continuous, geometrically open-ended process in which increasing shear progressively elongates and thins existing fibrils and generates additional fibril–fibril branching junctions so that the reinforcing network’s density and connectivity continue to increase up to the highest screw speed examined.

## Conclusions

The rheological modifications induced by HNC and PTFE significantly influenced cell nucleation and growth during microcellular foaming. The elevated tan δ of the PLA/HNC composite promoted rapid cell growth but increased the susceptibility to cell coalescence and collapse. In contrast, the enhanced elasticity and viscosity of the PLA/PTFE composite improved melt strength and strain hardening capacity, hindering cell merging and collapse.

Increasing screw speed enhanced shear-induced dispersion and nucleation efficiency. In PLA/HNC, deagglomerated HNC particles increased heterogeneous nucleation site density and reduced average cell size by approximately 50–60% between 20 and 120 rpm. Cellular morphology and void fraction (∼20.5% maximum) reached saturation above 60 rpm, beyond which further shear intensity yielded no additional microstructural refinement. Despite achieving lower ultimate void fractions than HNC, the PLA/PTFE system reached its plateau porosity at a lower critical screw speed, reflecting the lower shear energy threshold for PTFE fibrillation initiation relative to HNC deagglomeration. In the PLA/PTFE system, screw speed drove progressive in situ fibrillation of PTFE particles into an increasingly dense, topologically connected nanofibrillar network (∼100–500 nm fiber diameter).

The PLA/PTFE system achieved specific yield strength and specific toughness of ∼68–70 MPa·cm^3^/g and ∼11 J/g at 120 rpm, surpassing PLA/HNC maxima by ∼50% and ∼80%, respectively. This behavior, absent in the PLA/HNC system, which plateaued above 80 rpm, was directly attributed to the continuing densification and topological connectivity of the PTFE fibrillar network at high shear rates. The physically entangled PTFE fibrils serve a dual mechanical function: they reinforce the cell walls against buckling and fracture under compressive loading, and their fibrillar continuity across cell wall junctions provides crack bridging and energy dissipation mechanisms not available in the particulate HNC system. The strain-hardening character increasingly evident in PLA/PTFE reflects the alignment and tensile loading of PTFE fibrils during cell wall deformation. The substantially accelerated crystallization kinetics of the composites, particularly the ability of the PLA/PTFE composite to achieve near-complete crystallization within 1.6–2.5 min under isothermal conditions, contributed well to rapid cell wall solidification during foam extrusion and cooling, stabilizing the cellular architecture at an early stage of expansion and contributing to the fine, uniform morphology.

## Supplementary Material



## Notes

Apparent shear rates corresponding to the investigated screw speeds; Complex viscosity (η*) of PLA and its composites recorded as a function of temperature; DSC first heating thermograms of neat PLA, PLA/HNC, and PLA/PTFE composites; Storage modulus, loss modulus and tan δ of PLA and its composites recorded as a function of temperature; SEM micrograph of neat PLA foams at different screw speeds (PDF)
