# Supplementary material for: Shear-Induced Evolution of Cellular Structure and Thermo-Mechanical Properties in PLA/HNC and In-situ Fibrillated PLA/PTFE Composite Foams
Source: Langmuir. 2026 Aug 27;42(35):25899–911. doi: 10.1021/acs.langmuir.6c03508 (PMC13564964; doi:10.1021/acs.langmuir.6c03508)
Supplement: Supplementary file 1 [file la6c03508_si_001.pdf]

Supporting Information

Shear-Induced Evolution of Cellular Structure and Thermo-Mechanical Properties in PLA/HNC and In-situ Fibrillated PLA/PTFE Composite Foams

Silla George Raju[1,2*], Ramin Hosseinnezhad[2*], Andrzej Galeski[2]

[1] BioMedChem Doctoral School of the University of Lodz and Lodz Institutes of the Polish Academy of Sciences, 21/23 Matejki Street, 90-237 Lodz, Poland

[2] Centre of Molecular and Macromolecular Studies, Polish Academy of Sciences, Sienkiewicza 112, 90-363 Lodz, Poland

*Corresponding Authors: silla.george-raju@cbmm.lodz.pl, ramin.hosseinnezhad@cbmm.lodz.pl

Number of pages: 4
Number of figures: 4
Number of tables: 1

Table of Contents

Table S1. Apparent shear rates corresponding to the investigated screw speeds

Figure S1. Complex viscosity (η*) of PLA and its composites recorded as a function of temperature

Figure S2. DSC first heating thermograms of neat PLA, PLA/HNC, and PLA/PTFE composites

Figure S3. Storage modulus, loss modulus and tan δ of PLA and its composites recorded as a function of temperature at heating rate of 2 °C/min

Figure S4. SEM micrographs of neat PLA foamed at 20 rpm, 40 rpm, 60 rpm, 80 rpm, 100 rpm, and 120 rpm

Table S1. Apparent shear rates corresponding to the investigated screw speeds.

Screw speed (rpm)	Volumetric flow rate (cm^3/min)	Apparent shear rate (1/s)
20	0.17	74
40	0.20	126
60	0.27	168
80	0.33	210
100	0.40	252
120	0.47	294

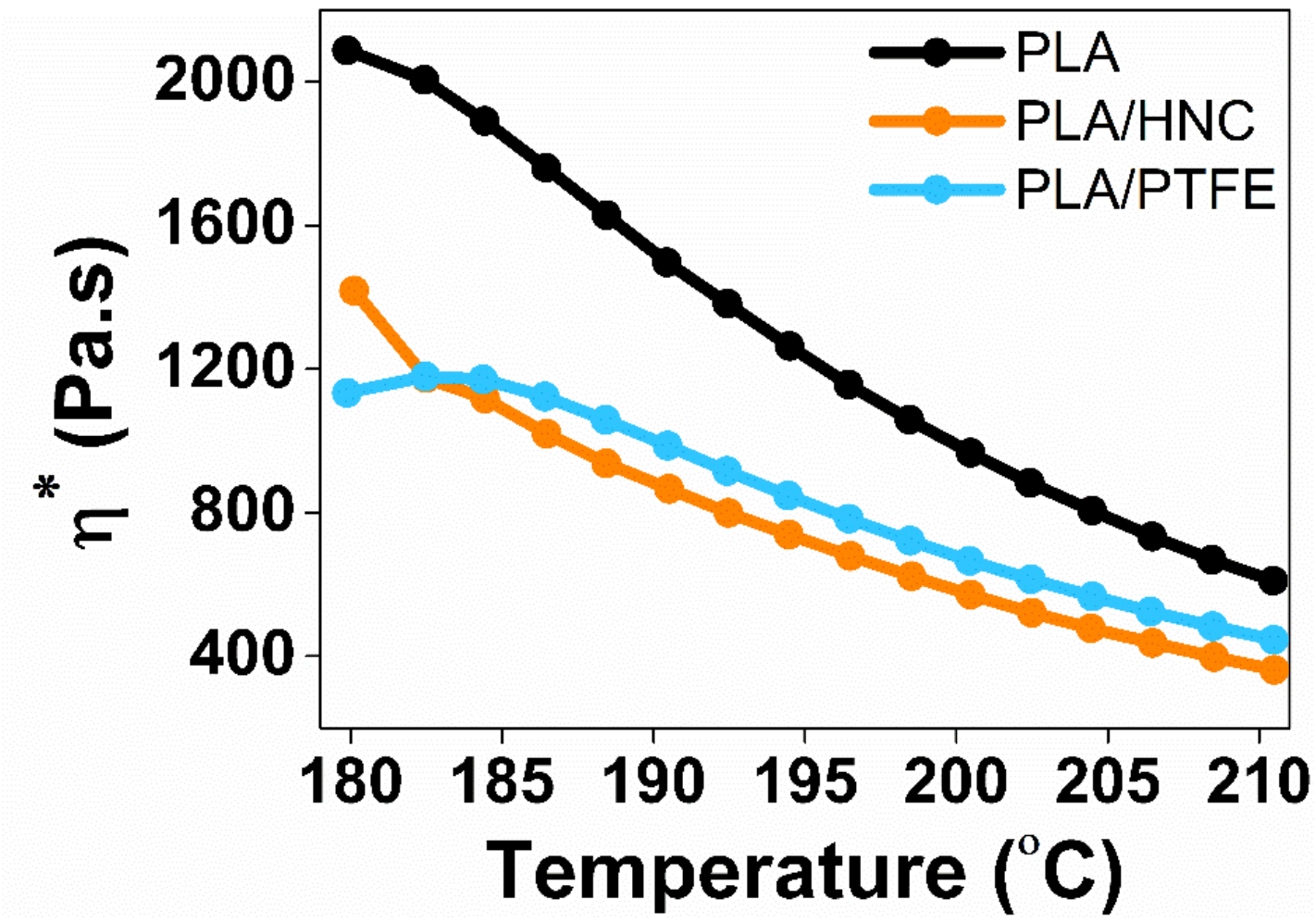

Figure S1. Complex viscosity (η*) of PLA and its composites recorded as a function of temperature.

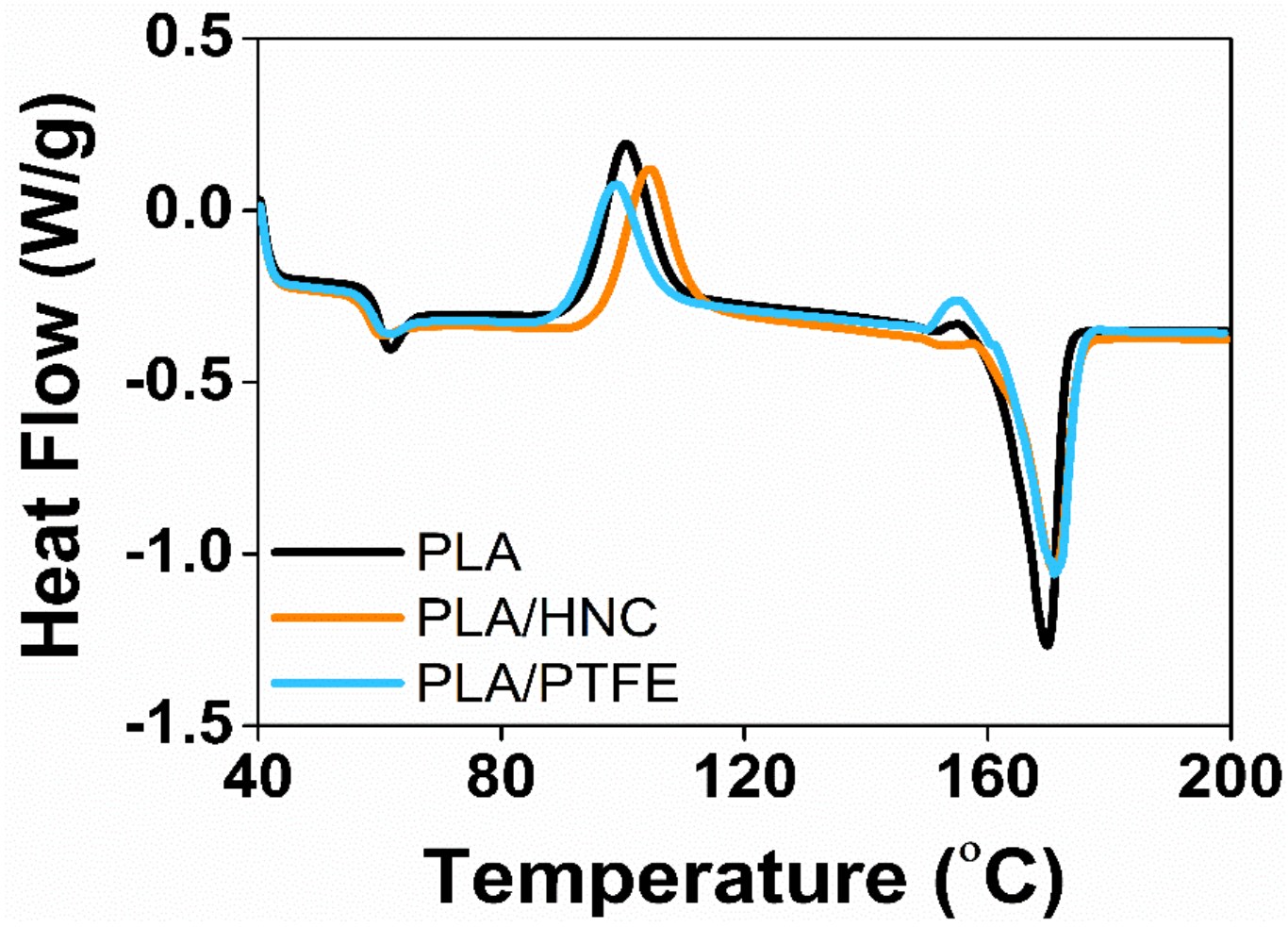

Figure S2. DSC first heating thermograms of neat PLA, PLA/HNC, and PLA/PTFE composites.

The solid-state dynamic mechanical response of neat PLA and its composites is shown in Figure S3. All materials exhibit a glassy storage modulus (G′) of ~3000-4000 MPa below T_g (60-65 °C). Above T_g, neat PLA softens to only a few MPa before partially recovering during cold crystallization, whereas both composites retain a significantly higher rubbery-plateau modulus, with PLA/PTFE showing the highest value. Since the tan δ peak at T_g is nearly unchanged, the enhanced rubbery stiffness is attributed to filler reinforcement rather than changes in segmental relaxation. The superior modulus of PLA/PTFE is consistent with its load-bearing fibrillar network, while the lower reinforcement of PLA/HNC reflects interfacial debonding around HNC particles, which limits stress transfer.

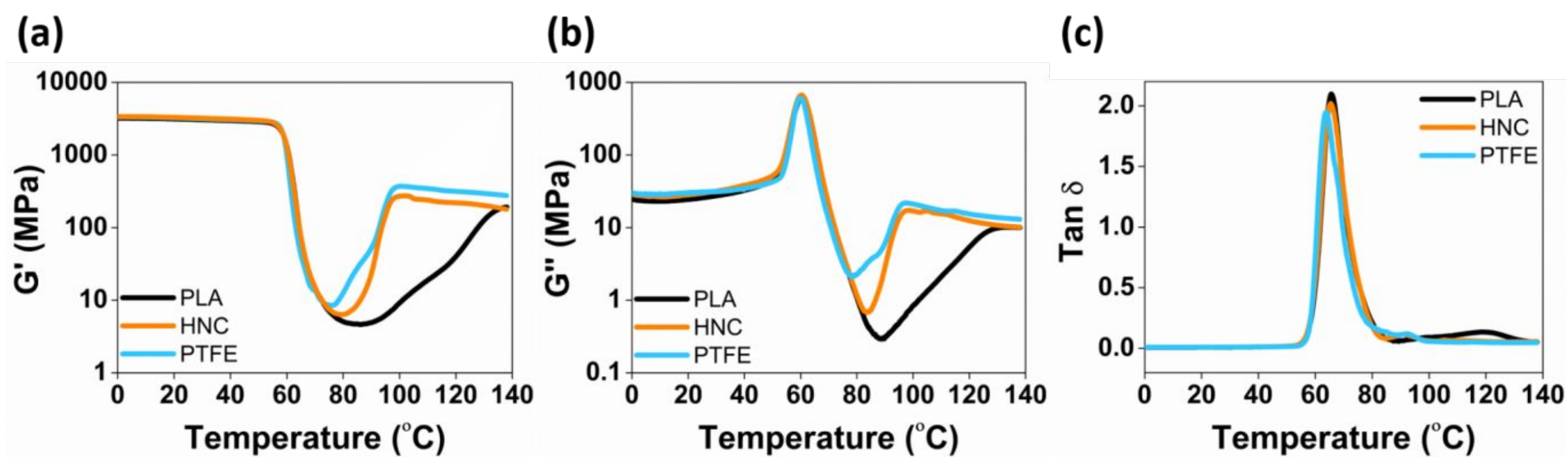

Figure S3. (a) Storage modulus, (b) loss modulus and (c) tan δ of PLA and its composites recorded as a function of temperature at heating rate of 2 °C/min.

Micrographs of neat PLA foams processed across the same screw-speed range, are given in Figure S4. At 20 rpm, neat PLA shows a coarse, highly heterogeneous structure with large, often irregular and locally coalesced cells, consistent with homogeneous nucleation from ADC decomposition products in the absence of any heterogeneous nucleating filler. Increasing screw speed produces only modest refinement. Cell size becomes somewhat more uniform, but even at 120 rpm the neat PLA foam retains a broader size distribution, a lower apparent cell density, and more frequent large, irregular cells than either PLA/HNC or PLA/PTFE processed at the same speed.

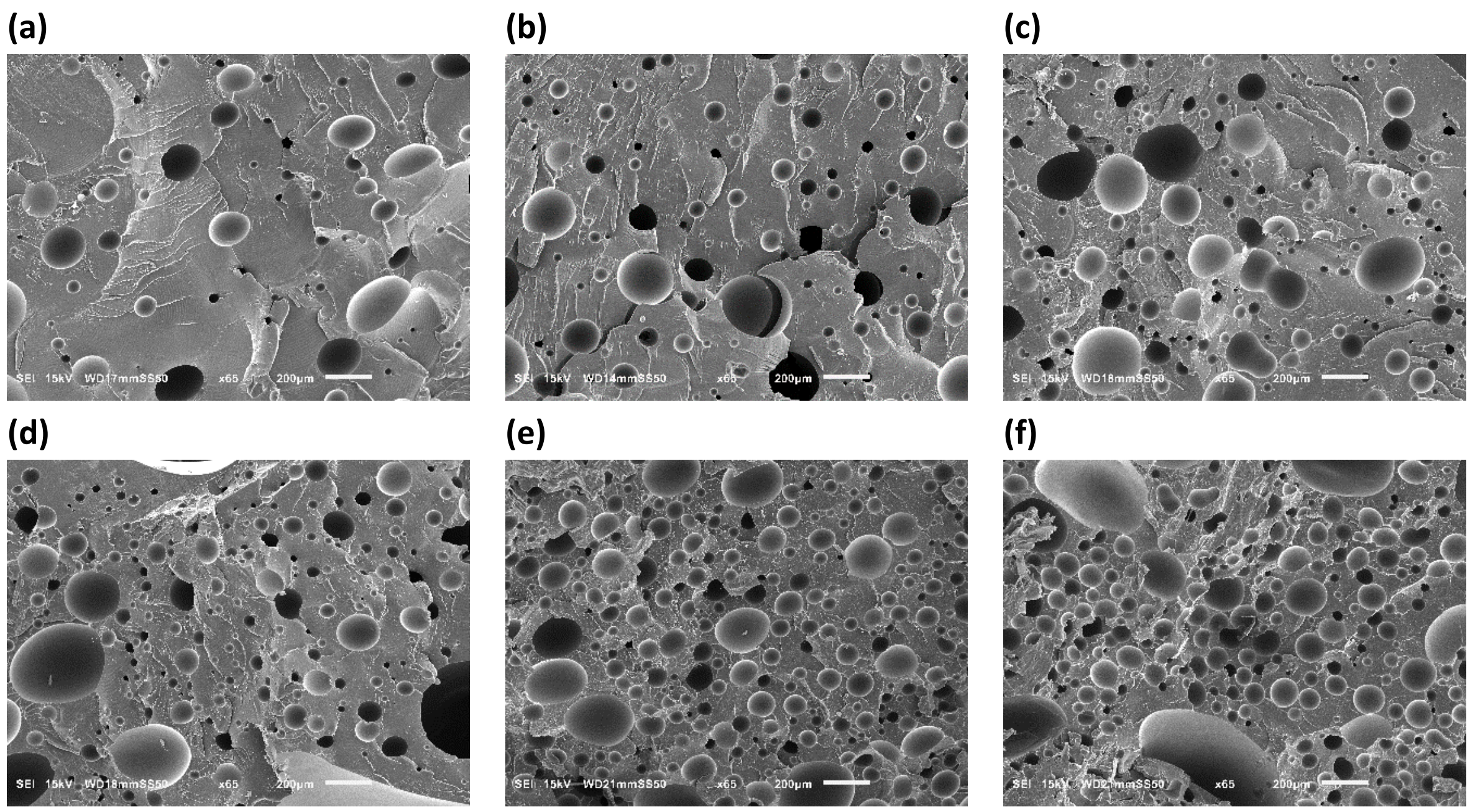

Figure S4. SEM micrographs of neat PLA foamed at (a) 20 rpm, (b) 40 rpm, (c) 60 rpm, (d) 80 rpm, (e) 100 rpm, and (f) 120 rpm. Scale bar: 200 µm.
